# ONION: Functional Approach for Integration of Lipidomics and Transcriptomics Data

**DOI:** 10.1371/journal.pone.0128854

**Published:** 2015-06-08

**Authors:** Monika Piwowar, Wiktor Jurkowski

**Affiliations:** 1 Department of Bioinformatics and Telemedicine, Jagiellonian University, Kopernika 7E, 31–062 Kraków, Poland; 2 The Genome Analysis Centre, Norwich Research Park, Norwich NR4 7UH, United Kingdom; National Institute of Genomic Medicine, MEXICO

## Abstract

To date, the massive quantity of data generated by high-throughput techniques has not yet met bioinformatics treatment required to make full use of it. This is partially due to a mismatch in experimental and analytical study design but primarily due to a lack of adequate analytical approaches. When integrating multiple data types e.g. transcriptomics and metabolomics, multidimensional statistical methods are currently the techniques of choice. Typical statistical approaches, such as canonical correlation analysis (CCA), that are applied to find associations between metabolites and genes are failing due to small numbers of observations (e.g. conditions, diet etc.) in comparison to data size (number of genes, metabolites). Modifications designed to cope with this issue are not ideal due to the need to add simulated data resulting in a lack of p-value computation or by pruning of variables hence losing potentially valid information. Instead, our approach makes use of verified or putative molecular interactions or functional association to guide analysis. The workflow includes dividing of data sets to reach the expected data structure, statistical analysis within groups and interpretation of results. By applying pathway and network analysis, data obtained by various platforms are grouped with moderate stringency to avoid functional bias. As a consequence CCA and other multivariate models can be applied to calculate robust statistics and provide easy to interpret associations between metabolites and genes to leverage understanding of metabolic response. Effective integration of lipidomics and transcriptomics is demonstrated on publically available murine nutrigenomics data sets. We are able to demonstrate that our approach improves detection of genes related to lipid metabolism, in comparison to applying statistics alone. This is measured by increased percentage of explained variance (95% vs. 75–80%) and by identifying new metabolite-gene associations related to lipid metabolism.

## Introduction

In recent years, research is becoming increasingly focused on the widest possible inclusion of biological processes at the level of cells and tissues from one and multiple organisms (e.g. the effect of bacterial flora on human metabolic processes). The huge amounts of data produced with high-throughput techniques make the information and knowledge harvesting challenging for technical and interpretation reasons [1,2]. Integration of data on multiple levels gives the opportunity for filtering high quality molecular signals and unravels biological complexity in unprecedented way.

Multivariate statistics are often used to explain complex relationships in the data. Typically in e.g. clinical applications number of observations is larger than explained features. In the case of high-throughput data, thousands of variables (e.g. expression of multiple transcription variants) are matched with simultaneous low numbers of observations (specific conditions, biological replicates, time points). In this respect, modifications of classic methods are needed to circumvent technical issues or to improve the predictive performance, e.g. regulated Canonical Correlation Analysis (rCCA) [3] or sparse Partial Least Square regression (sPLS) [4] to name just few. The objective of these approaches is the reduction of variables, with the final set of variables included in the model narrowed down in a mathematically justified way (with specific assumptions). Application of the described methods in analysis of e.g. medical data, provides results which may be of major importance in drawing conclusions significant from the clinical viewpoint [5].

This paper presents a novel procedure to support interpretation of orchestrated changes in cellular metabolism and gene expression. Its main objective is to reduce initial data size by creating groups, providing molecular functions are known, in order to reach feasibility of statistical analyses. The functional constraints specified (e.g. ontology terms, molecular interactions) are weak to avoid skewing towards highly specific biological pathways. The presented methodology may be applied for various data sets, and for lipidomics and transcriptomics in particular. The applicability has been illustrated on the example of murine nutrigenomics data [6] by identification of important genes associated with lipid metabolism.

## Materials and Methods

### Lipidomics and transcriptomics integration workflow

**Fig 1:**
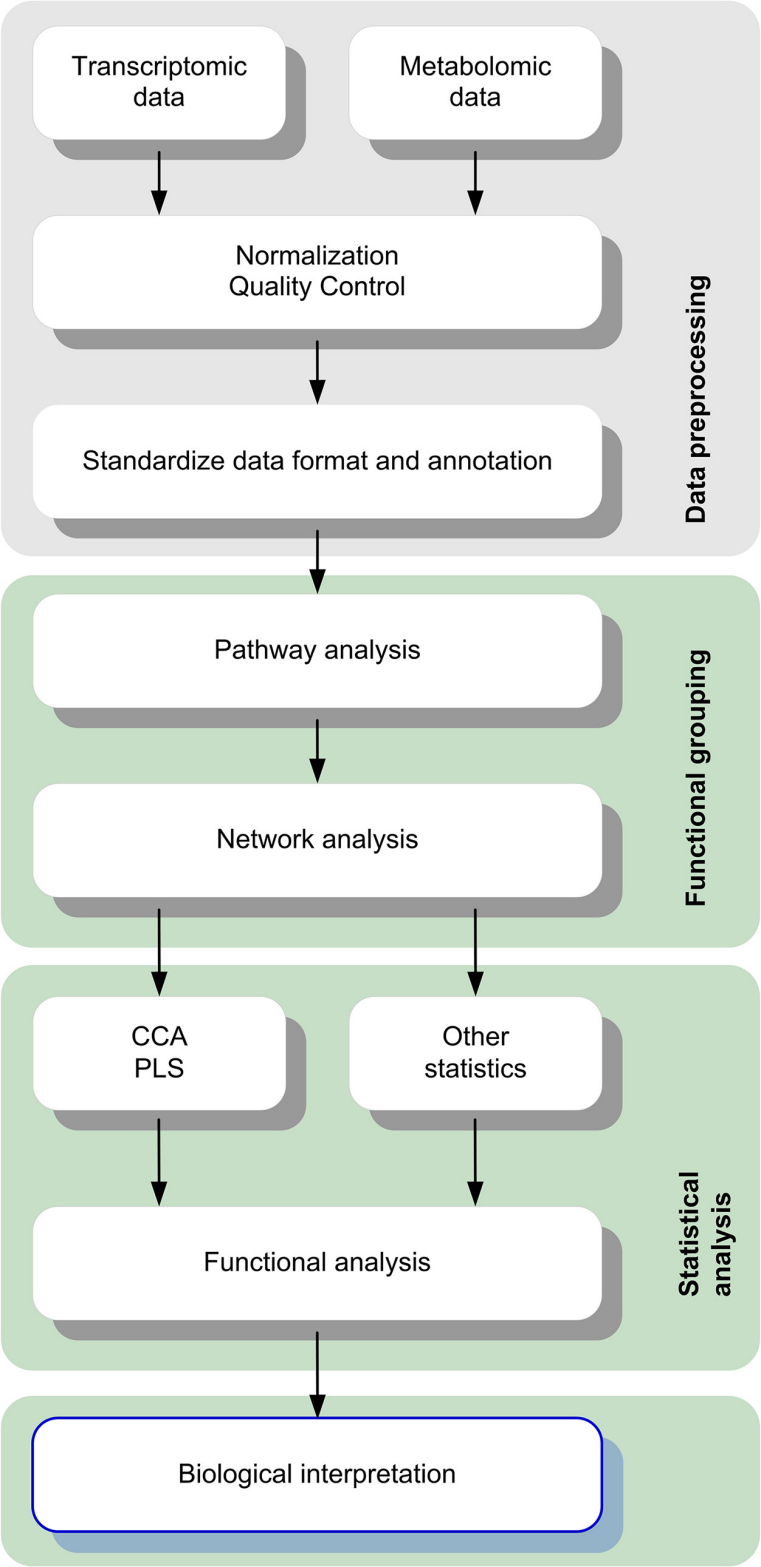
Workflow of metabolomics and transcriptomics data integration. After preprocessing, transcriptomics and metabolomics data are used to divide data set into functional groups. CCA and PLS statistics are calculated for each group in question to find rankings of gene–small molecule associations. Subsequent validation and functional analysis (e.g. by overrepresentation tests) helps in biological interpretation of ranked results.

The lipidomics and transcriptomics integration workflow is presented in Fig 1 to illustrate all necessary steps from raw data processing to functional interpretation. It comprises data preparation, identification of lipids and genes groups and statistical tests to find significant associations between genes and metabolites. The workflow prototype is implemented in bash and R and available at https://bitbucket.org/VHG-IG/onion. We do not intend to cover vast possibilities for data normalisation or calling differential expression and metabolite detection to name just a few. Although, we leave it to the user to apply the strategy most appropriate to a particular scenario, we would like to stress the need to provide standardized data annotations.

#### Data pre-processing

Data used for the analysis should meet the basic criteria of quality and be previously normalized with approaches appropriate to specific use cases. For instance we used LOESS regression implemented in agilp package [7] to normalize Agilent microarray data. Standardisation of nomenclature is based on Lipid Maps (http://www.lipidmaps.org/) and ChEBI (https://www.ebi.ac.uk/chebi/) in case of small molecules, and Ensembl (http://www.ensembl.org) or RefSeq (http://www.ncbi.nlm.nih.gov/refseq/) are resources to control proper mRNA variant naming. Correct handling of the naming scheme is crucial for tracking of heterogeneous data derived from multiple technological platforms and primary analysis software to deliver proper annotation standards for subsequent steps. Despite existence of data specific ontologies and annotation standards such as provided by Lipid Maps, multiple cases across scientific literature evidently show that these standards are not yet widely adopted. This results in molecules escaping automated handling and manual curation is required.

#### Functional groups of genes and lipids

Initial sets of genes involved in metabolic processes of fatty acids and lipids were found by searching Reactome pathways. Reactome is developed as an international collaboration aiming at the widest and most coherent description of cellular pathways in multiple model species [8]. First, for a given list of small molecule ChEBI identifiers [9] we find reactions in which these molecules participate either as substrates or products. Then, the Ensembl gene identifiers [10] are translated to match the identifier type present in the analysed expression data. This step utilises BioMart (www.biomart.org) [11] available in R through the biomaRt library [12]. Next, a set of differentially expressed genes is used to build a query in the STRING database via the STRINGdb Bioconductor library [13] to find interacting proteins and associations between genes. We use topological analysis such as distance from the lipid metabolism genes, co-occurrence in directed circuits and hub detection to expand the initial list of genes often not directly associated with lipid functions. Evidences such as text mining results, protein-protein interactions and gene proximity are taken into consideration on the condition of high quality of information from at least one source. Topological analysis is made with igraph library [14]. At the end we obtain a broad list of potential interactions to be sifted later by statistical testing with two goals: to minimise potential bias towards well-characterised biochemical pathways and at the same time propose new putative links relevant for the specific study (tissue, conditions).

#### Testing significant associations

Canonical Correlation (CCA) as well as regularized Canonical Correlation (rCCA) was applied for assessment of associations between two groups of variables: lipidomics (responses) and transcriptomics (predictors). Canonical correlation can be applied iteratively to identify a set of prediction variables explaining the largest range of variability of a set of responses. In addition, elimination of highly correlated variables is the condition for correct application of canonical analysis. That is why prior to the proper canonical analysis, variables correlated above a certain threshold (e.g. r>0.7) are temporarily removed from the analysed data sets.

Depending on the data structure, the relevant type of canonical analysis should be selected. Classical CCA cannot be applied to the entire data set due to its structure (110 transcriptomics and 41 lipidomics variables with only 40 observations), therefore this approach was used only for analysis within functional groups. We used CCA implemented in R package yacca [15]. To compare performance of both classic and regularized approaches we applied rCCA implemented in FRCC [16] for both groups and the undivided data set. For the interpretation we focus mainly on the canonical correlations between pairs of first two canonical variables (CV1 and CV2) and structural correlations (loadings) between data sets and their respective canonical variables.

Apart from the Canonical Analysis (CCA and rCCA), Partial Least Squares Analysis (PLS and sPLS) is also efficient for data integration and assessment of the relationship of two groups of variables [17]. The aim of PLS is to predict or analyse a set of dependent variables from a set of independent variables or predictors [18,19] based on calculation of latent variables (LV). PLS is used routinely in exploration analysis, to select convenient predictors and to identify deviated observations. The practical difference between application of canonical analysis and PLS consists mostly in different preliminary premises. In PLS, filtering of correlated dependent and independent variables and specific structure of the data are no longer required for technical reasons yet approaches to select variables like sPLS are used to improve prediction performance affected by the size of linear combinations. First latent variable (LV1) as well as loadings (regression coefficients) for LV1 were considered to interpret associations between genes and metabolites. On the other hand, unlike in CCA, loadings of individual variables obtained in PLS are less suitable to pinpoint contributions of individual observations when number of variables is much larger than samples [20].

Finally, in order to compare results obtained by different techniques we may compare rankings of genes or association pairs rather than actual statistics of individual methods. We are then testing the number of new hits that: 1) could not be identified by looking at separate data sets alone; and 2) share functions relevant from the standpoint of metabolic priors. Furthermore, functions not previously implicated in the biological context in question could indicate interesting candidates for elucidation of new connections between metabolites or genes.

#### Comparison with simulated data sets

An important aspect of interpretation of results obtained for groups and the undivided data set is the randomness of group composition. To assess the robustness of results obtained for specific groups we tested whether the levels of correlation or percentage of explained variance could be obtained merely by chance (H0). We simulated data sets by drawing lipids and genes in the same proportions as in the group in question in order to provide comparable conditions. For each such group we applied CCA and PLS and reported the fraction of given variables explained (Aggregate Redundancy Coefficients) and the percentage of explained variance respectively. We repeated this procedure 1000 times and compared the value yielded by our workflow with the mean values for each of the randomized data sets, testing significance with a one-sample t-test.

## Results and Discussion

### Performance on grouped and undivided data

We demonstrate our approach on data from a murine nutrigenomics study [6] available via the mixOmics R package. The population comprises mice nurtured in five different diet regimes, with 40 individuals in total. Based on the analysis of hepatic samples (four biological replicates at three time points each) it comprises gene expression data of 120 selected genes potentially involved in lipid metabolism and concentrations of 21 fatty acids. The data required treatment to match current gene symbol (HUGO) and small molecule nomenclatures (ChEBI). In a few cases gene symbols were not mapped precisely and we worked with a set of 110 genes in total. See *Complete lists of canonical variables* (S1 Data) for a full list of mapped identifiers. As raw data is not available, other pre-processing steps were not needed. Groups are created starting from the primary division of fatty acids to saturated mono and polyunsaturated, through associations of genes from relevant pathways and finally by expansion of the groups by topological analysis of the STRING interactome. Group 4 consists of metabolites and genes not included in Groups 1–3.

Canonical analysis techniques are applied to the undivided data set as well as functional groups (Table 1). Although, available data sets are relatively small (110 genes and 21 lipids), they are still too large for given number of samples (40 points) in order to successfully apply classical canonical correlation and we supplemented it with rCCA. The analysis reveals associations that are weaker in the undivided data (CV1 = 0.89, CV2 = 0.78) than in any of the groups (Table 1). In addition rCCA is subject to shortcomings: simulations are required to increase the size of the data, with a consequent lack of statistical significance test. For comparison, PLS was applied for the functional groups and undivided data sets as well. Results revealed that using functional groups (in all groups except Group1) leads to improvement in finding significant associations measured by percentage of explained variance.

**Table 1:** Results of Canonical Correlation Analysis of murine nutrigenomics.

	Group 1	Group 2	Group 3	Group 4	Undivided
**X** r>0.7/all	20/51	22/65	22/65	19/45	120
**Y** r>0.7/all	2/2	6/6	2/2	7/11	21
**CCA** CV1	0.95 (p = 0.4e-03)	0.98 (p = 1.8e-06)	0.92 (p = 2e-04)	0.95 (p = 8.2e-05)	-
**CCA** CV2	0.74 (p>0.5)	0.95 (p = 5.1e-03)	0.88 (p = 1.5e-02)	0.93 (p = 0.007)	-
**Redundancy X | Y**	0.10	0.32	0.14	0.35	-
**Redundancy** Y | X	0.85	0.74	0.82	0.65	-
**rCCA** CV1	0.97	0.986	0.98	0.984	0.89
**rCCA** CV2	0.87	0.984	0.96	0.966	0.78
**PLS** LV1	19.07	37.55	36.91	30.83	33.46
**PLS** LV2	15.02	13.15	16.74	30.37	18.33

Number of genes (X) and metabolites (Y) in each group: after removing correlated r>0.7 variables and all. Correlations of Canonical Variables (CV1 and CV2), aggregate redundancies, percentage of variance explained by PLS latent variables are calculated within functional groups (Group 1–3), remaining variables (Group 4) and for undivided data set.

Functional analysis of top ranked solutions can be used to directly compare methods using different metrics as PLS or CCA. We counted the number of new lipid/fatty acid–gene associations not originally given by interrogating the sets of biochemical reactions directly involving the fatty acids in question. At the same time we can test for bias caused by using knowledge in the initial group definition. In order to do that we looked at the top 10% of gene candidates selected by CCA or PLS and checked their presence in: reactions queried by lipids/fatty acids defining group assignment; Reactome knowledge base; and in all pathways involving lipids. We present these counts in Table 2 together with lists of significantly enriched pathways (Table 3). The first important observation is the presence of minimal bias in group 1 (2 genes) and group 2 (1 gene). These are the only genes coding proteins involved in biochemical reactions of lipids or regulated by fatty acids (PPARA) and were selected from within the Reactome knowledge base by metabolomics results. Interestingly, top candidates found in the full data set are less frequently associated with the broad category of lipid metabolism. Thanks to interactome based selection we were able to highlight genes involved in lipid metabolism that would have been missed if analysing the transcriptomics data alone. For each category (except the full data set) there is number of significantly enriched pathways that are interesting from the perspective of both metabolism and role of lipids in cellular processes such as the regulation of gene transcription. The list of top ranked genes and details of PLS loadings which the ranking was derived from are given in *List of top genes* (S1 Table).

**Table 2:** Top 10% ranked PLS results in respective categories/groups.

Group	In reactions involving detected lipids	In lipid pathways	Not present in Reactome
**Undivided**	0	3	3
**Group 1**	2 (APOA1, APOB)	7	1
**Group 2**	1 (PPARA)	8	2
**Group 3**	0	6	2
**Group 4**	0	2	5

We compare number of genes present in sets of seed biochemical reactions with other lipid metabolism and function related genes found by CCA. Last column provides lists of pathways significantly enriched within groups of genes.

**Table 3:** Pathways significantly enriched (FDR < 0.05) within groups of genes in top 10% ranked PLS results in respective categories/groups.

Group	Reactome ID	Pathway name	FDR
Undivided	-	-	-
**Group 1**	REACT_15525	"Nuclear Receptor transcription pathway"	1.2E-12
	REACT_12627	"Generic Transcription Pathway"	4.1E-5
	REACT_6823	"Lipoprotein metabolism"	4.1E-5
	REACT_13621	"HDL-mediated lipid transport"	3.7E-4
	REACT_163679,	"Scavenging by Class B Receptors"	0.0020
	REACT_602	"Lipid digestion, mobilization and transport"	0.0068
	REACT_22258	"Metabolism of lipids and lipoproteins"	0.012
	REACT_6841	"Chylomicron-mediated lipid transport"	0.012
	REACT_163699	"Scavenging by Class A Receptors"	0.019
**Group 2**	REACT_22258	"Metabolism of lipids and lipoproteins"	5.4E-4
	REACT_268803	"Defective CYP24A1 causes Hypercalcemia, infantile (HCAI)"	0.0018
	REACT_23947	"GABA synthesis, release, reuptake and degradation "	0.0023
	REACT_602	"Lipid digestion, mobilization and transport"	0.0068
	REACT_11082	"Import of palmitoyl-CoA into the mitochondrial matrix"	0.011
	REACT_22279	"Fatty acid, triacylglycerol, and ketone body metabolism"	0.023
	REACT_116145	"PPARA activates gene expression"	0.029
	REACT_118659	"RORA activates gene expression"	0.029
	REACT_19241	"Regulation of lipid metabolism by Peroxisome proliferator-activated receptor alpha (PPARalpha)"	,0.029
	REACT_147904	"Activation of gene expression by SREBF (SREBP)"	0.046
	REACT_264212	"Transcriptional activation of mitochondrial biogenesis"	0.046
	REACT_267785	"Signaling by Retinoic Acid"	0.046
	REACT_1190	"Triglyceride Biosynthesis"	0.049
**Group 3**	REACT_268803	"Defective CYP24A1 causes Hypercalcemia, infantile (HCAI)"	0.0016
	REACT_11082	"Import of palmitoyl-CoA into the mitochondrial matrix"	0.012
	REACT_22258	"Metabolism of lipids and lipoproteins"	0.012
	REACT_11042	"Recycling of bile acids and salts"	0.013
	REACT_11040	"Bile acid and bile salt metabolism",	0.049
	REACT_267785	"Signaling by Retinoic Acid"	0.049
**Group 4**	REACT_115639	"Sulfur amino acid metabolism"	0.019
	REACT_22279	"Fatty acid, triacylglycerol, and ketone body metabolism"	0.023
	REACT_163862	"Cobalamin (Cbl, vitamin B12) transport and metabolism"	0.024
	REACT_115589	"Cysteine formation from homocysteine",	0.032
	REACT_169149	"Defective MTR causes methylmalonic aciduria and homocystinuria type cblG"	0.032
	REACT_169439	"Defective MTRR causes methylmalonic aciduria and homocystinuria type cblE"	0.032

**Fig 2:**
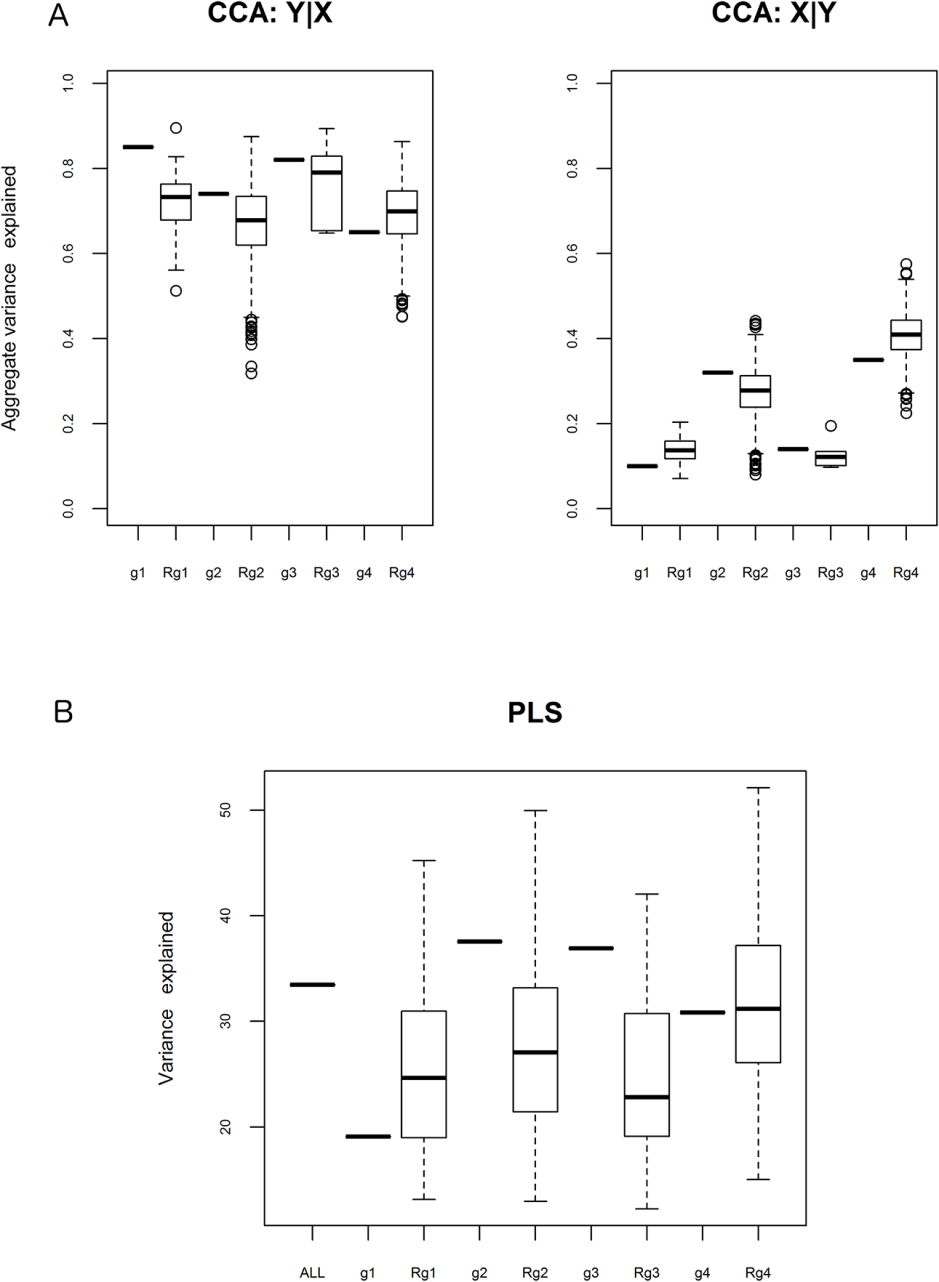
Comparison of randomized groups with (A) CCA and (B) PLS results. (A) CCA: Aggregate variability explained for Y|X and X|Y (B) PLS: % of explained variance. All differences between respective groups are significant (p <0.05, one-sample t-test). All–undivided data set; g1, g2, g3 –functional groups; g4 –complement; Rg1, Rg2, Rg3, Rg4 –mean values in randomized groups

The final consideration of this section is randomness of estimated associations. For each of the randomized groups (rg1, rg2, rg3 and rg4) we calculated the mean value of aggregated explained variability (CCA) and mean value of the percentage of explained variation (PLS). The results were compared with the values obtained with actual functional groups g1, g2, g3 and their complement g4. Results of PLS and CCA are concordant. The calculated aggregate variances and percentage of variance explained in each group are significantly different than in corresponding randomized groups (Fig 2) proving a lack of randomness in our results.

### Functional analysis

**Fig 3:**
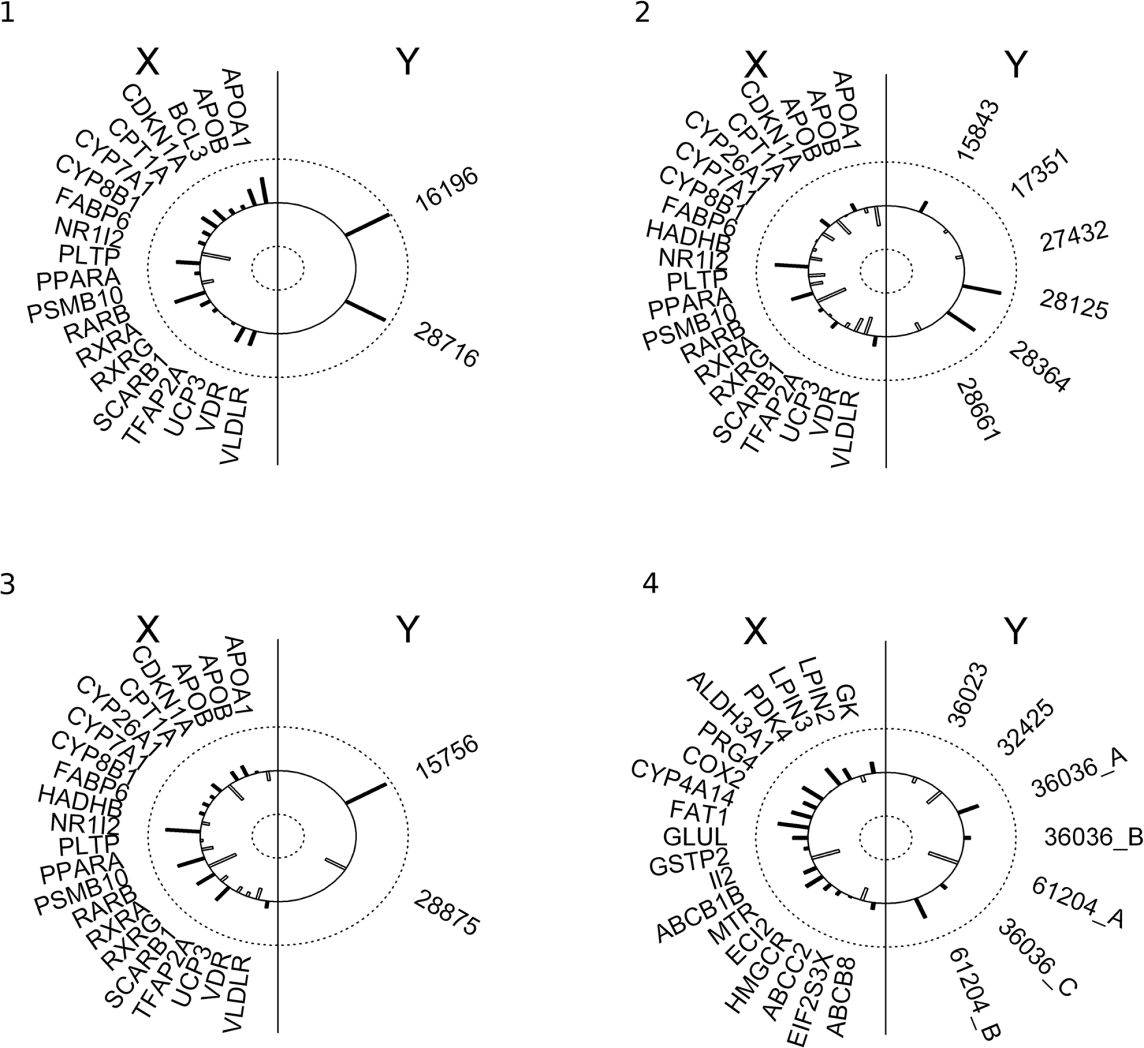
Graphical presentation of the first canonical correlation for each group (1–4). Correlation structure is indicated by length of the bars extending toward the circumference (positive correlation) or toward the centre (negative correlations). The left semicircle lists the transcriptional variables (X) and the right semicircle lists the fatty acids variables (Y).

Canonical analysis provides information about the scope of the explained variation in sets of lipidomics data (Y) based on transcriptomics data (X) and vice versa. First the canonical variable is statistically significant and very high (>0.9) across groups 1–4 suggesting high strength of associations between respective fatty acids and gene products. For instance, in group 1 oleic acid (ChEBI: 16196) and palmitoleic acid (ChEBI: 28716) are highly associated with RARB (retinoic acid receptor, beta), PLTP (phospholipid transfer protein), NR1I2 (nuclear receptor subfamily 1, group I, member 2) and APOA1 (apolipoprotein A-I). As APOA1 is directly involved in metabolism of fatty acids, it is an obvious candidate. The remaining genes are interesting hits due to participation in processes regulating or dependent on fatty acid regulated transcription and would have been overlooked with a typical pathway analysis. RARB stimulates hepatic induction of fibroblast growth factor 21 to promote fatty acid oxidation [21], PLTP has impact on metabolism of high-density lipoproteins [22] and transports diacylglycerol, and other molecules closely related with fatty acids metabolism. NR1I2 is a transcription factor binding to retinoic acid receptor RXR-RAR required by PPARG, involved in transcription regulation of multiple metabolic enzymes also implicated in regulation of diet-dependent metabolic syndrome [23] and regulates cytochrome P450 (CYP3A4) key to lipid synthesis. Finally, two genes LPIN1 and FABP6 are highly correlated to NR1I2 and PLTP respectively. LPIN1 regulates synthesis of triglyceride [24] and FABP6 is a bile and fatty acid transporter [25]. Similarly, in groups 2 and 3, genes that have the highest share in a given canonical variable (and then canonical correlations) are significantly involved in the metabolism of fatty acids: RARB, APOA1 and NR1I2 are already mentioned above. PSMB10 (proteasome subunit, beta type 10) is one of the proteasome components. It was recently shown that proteasome inhibition treatment caused down-regulation of multiple lipid metabolism enzymes causing subsequent decrease of fatty acid synthesis [26]. Lipids from group 2 with the largest contribution to canonical variables are: icosapentaenoic acid (ChEBI: 28364), and hexaenoic acid (ChEBI: 28125) (Fig 3). Similarly, in group 3, in which genes: RARB, CPT1A, RXRA (together with highly correlated CYP27B1, LDLR, LPIN1, NOS2, PPARD, SCARB1, UCP2, UCP3 and WDR) and PSMB10 share the largest association to palmitic (ChEBI: 15756) and myristic acids (ChEBI: 28875). CPT1A (carnitine palmitoyltransferase 1a) catalyses the primary regulated step in overall mitochondrial fatty acid oxidation [27] and CYP27B1, as other cytochrome P450 subunits, is involved in steroids synthesis.

Group 4 included genes not included in groups 1–3. Albeit, comparably high canonical variables are found also in this group, genes with the highest correlations have no known function dependent on or involved in lipid metabolism: FAT1 (FAT tumour suppressor homolog 1), PRG4 (proteoglycan 4), II2 (interleukin 2) suggesting spurious characterisation of these associations. Thus, groups 1 and group 4 vary by qualitative differences in biological interpretation of statistical result. In addition functional grouping finds genes having significantly higher impact on metabolites (higher redundancy) than in the case of undivided data AND allows best explanation of variability of Y by X. In group 1 Y|X redundancy is 0.85 (85% of variation in lipidomics data set is explained by transcriptomics data set) compared to 0.65 in group 4. The reverted relations are much weaker (from 0.10 to 0.35) due to an unbalanced number of genes and metabolites specific to the nutrigenomics data set in question (Table 4).

**Table 4:** The CCA and rCCA results for Groups 1–4 and undivided data set.

	Gene symbol	CCA loadings	rCCA loadings	ChEBI ID	CCA loadings	rCCA loadings
**Undivided**	SSX2IP	-	0,77	32425	-	-0,45
	THRSP	-	0,57	35465	-	-0,43
	LPIN1	-	0,46	36036	-	1,06
	CYP24A1	-	0,43	-	-	-
**Group 1**	NR1I2/*LPIN1*	-0,55	0,08	16196	0,96	0,65E-3
	APOA1	0,59	-0,043	28716	0,87	-0,17
	RARB	0,62	-0,16	-	-	-
	PLTP/*FABP6*	0,45	-0,023	-	-	-
**Group 2**	RARB	-0,61	-0,16	28364	0,66	0,12
	APOA1	-0,44	-0,043	28125	0,75	0,12
	PSMB10	0,43	0,25	-	-	-
	NR1I2	0,64	0,080	-	-	-
**Group 3**	RARB	-0,58	-0,16	15756	0,89	0,05
	CPT1A	-0,42	-0,11	28875	-0,45	0,05
	RXRA/ *CYP27B1*, *LDLR*, *LPIN1*, *NOS2*, *PPARD*, *SCARB1*, *UCP2*, *UCP3*, *VDR*	0,40	0,12	-	-	-
	PSMB10	0,54	0,25	-	-	-
	NR1I2	0,67	0,080	-	-	-
**Group 4**	FAT1, *COX1*	0,59	0,18	61204_A	-0,60	-0,16
	PRG4	0,46	0,11	61204_B	0.47	0,12
	PDK4	0,45	0,12	36036_A	0.41	1,06
	Il2	-0,55	-0,19	-	-	-

For both metabolites and genes canonical loadings describe the impact of particular variables on correlations between data sets. Gene symbols in italic are correlated (r>0.7) with variables in question and were removed prior canonical analysis.

The size of the undivided data set does not allow the calculation of CCA and only rCCA can be practically applied resulting in significantly lower correlations (for λ1 = 0.064, λ2 = 0.008) than in groups 1–4 (Table 4). Most importantly, due to the lack of significance test, the validity of associations can be questioned allowing only approximate ranking of solutions. This creates difficulty in interpreting relationships between the data X|Y and Y|X similar to that obtained in CCA. In addition, low weights of variables that constitute canonical correlations do not allow finding many associations of particular genes and fatty acids. The highest associations were found for the following genes: SSX2IP (synovial sarcoma, X breakpoint 2 interacting protein), THRSP (thyroid hormone responsive), LPIN1 (lipin 1) and CYP24A1 (cytochrome P450, family 24, subfamily A, polypeptide 1) with fatty acids: gondoic acid (ChEBI: 32425), (Z)-hexadec-7-enoic acid (35465), (ChEBI: icosatrienoic acid). The highest weight is assigned to SSX2IP that amongst the others plays a role in adherens junctions and cell adhesion [28] and is not related to fatty acid or lipid metabolism.

## Discussion and Conclusions

The methods used in integration of biological data differently account for biological context and emphasise interpretation of individual data types. In some cases the relation between different levels are function-agnostic and the test probabilities obtained from independent experiments are used to determine ranks of the solutions [29,30] in the others multiple data types are synergistically merged together to develop models tailored to specific biological phenomena e.g. data-driven network contextualization to study functional states [31]. Statistical techniques like CCA or rCCA do not include the biological context of the analysed variables. Despite further modifications of underlying statistical models [32] the typical outcome is still not satisfactory and the results can be difficult to interpret.

Biological function is most often taken into account by including pathway and gene ontology analysis, e.g. for finding genes involved in metabolic processes [33] or to analyse expression changes [34]. Network analysis defines alternative paths between phenotypes (gene expressions) with genotypes, which are then compared in terms of optimum explanation of numerous possible perturbations (e.g. mutations) [35]. Thus, considering the fact that genes, proteins and other molecules do not operate autonomously but are mutually dependent in continuously changing processes, incorporating biological priors into the analysis is substantively justified.

Herein, we present a novel procedure that effectively applies multivariate statistics for large high-throughput data sets making use of molecular interaction networks to guide selection of variables. It is worth mentioning that multivariate statistics implemented specifically for omics-data integration are available and widely used, including R packages mixOmics [36]. We find our approach a valuable complement, because 1) it presents strategy for gradual analysis of all variables rather than excluding any variables a priori; 2) the number of observations and variables are balanced without modification of original matrix by adding or removing data to avoid matrix singularity. Application to murine nutrigenomics data reveals strong associations between genes and fatty acids including previously unreported links involved in lipid metabolism.

The main limitation of the approach is relying on strong biological priors, in the form of reliable molecular networks, to source functional relationship between entities. Albeit, the potential bias is small and is limited to initial sets of genes coding proteins acting directly on metabolites under study it requires attention to ensure that local network connectivity is sufficient. This can be controlled in practical use scenario by looking at sizes and numbers of groups generated relative to the initial data size. In addition, at this stage the workflow is only suited to study data of well-annotated species such as mice and human; in the future, we plan to extend the approach by testing limits of orthologous annotation to guide the data division into functional groups.

## Supporting Information

S1 Data Tabular data in Excel format. Complete lists of canonical variables. Supplementary data set lists all canonical variables identified as well as highly correlated (>0.7) genes and metabolites excluded prior analysis. Each sheet corresponds to either metabolite or gene data of particular group.
(XLSX)

S1 Table Tabular data in Word format. List of top genes. In addition to counts presented in Table 2 this supplementary table lists symbols of top 10% of genes according to PLS and CCA rankings.
(DOCX)
