# Supplementary material for: ONION: Functional Approach for Integration of Lipidomics and Transcriptomics Data
Source: PLoS One. 2015 Jun 8;10(6):e0128854. doi: 10.1371/journal.pone.0128854 (PMC4459700; doi:10.1371/journal.pone.0128854)
Supplement: S1 Table — List of top genes. In addition to counts presented in Table 2 this supplementary table lists symbols of top 10% of genes according to PLS and CCA rankings. (DOCX) [file pone.0128854.s002.docx]

Supplementary figure 1.

Results of data integration with PLA, CCA and rCCA. The table provides highest loading values for each of the groups: *g1, g2, g3, g4* and undivided data set.

|  | **PLS** | | | | **rCCA for undivided data CCA for groups** | | | |
| --- | --- | --- | --- | --- | --- | --- | --- | --- |
|  | **X**  **Gene symbol; loadings** | | **Y**  **Lipid ID; loadings** | | **X**  **Gene symbol; loadings** | | **Y**  **Lipid ID; loadings** | |
| **All** | **SSX2IP**  **THRSP**  **CYP24A1**  **LPIN1**  **DBI**  **ECI2**  **SLC10A1**  **RARB**  **PON1**  **Il2**  **CIDEA**  **MTHFR** | **0.41**  **0.32**  **0.26**  **0.23**  **0.20**  **0.18**  **0.01**  **0.02**  **0.03**  **0.03**  **0.04**  **0.12** | **15756**  **28125**  **28842**  **36023**  **15843**  **28364**  **35465**  **32425**  **61205**  **72850**  **27432**  **17351** | **4.67**  **2.08**  **1.53**  **1.28**  **0.91**  **0.66**  **0.09**  **0.12**  **0.13**  **0.20**  **1.83**  **10.31** | **ACACB APOA1 PPARA ECI2 FABP2 NOS2 VLDLR RARB Il2 PSMB10 NR1I2 PRG4** | **0.21**  **0.23**  **0.25**  **0.28**  **0.31**  **0.31**  **0.41**  **0.53**  **0.56**  **0.57**  **0.57**  **0.65** | **61204 32425 36036 28661 15756 36023 28364 28125** | **0.55**  **0.28**  **0.34**  **0.36**  **0.52**  **0.64**  **0.75**  **0.86** |
| **g1** | **APOB  CYP7A1  RARB  FABP2  PLTP  APOA1**  **FAS**  **NR1H3  NR1I2  PSMB10  LPIN1**  **DBI** | **0.30**  **0.30**  **0.29**  **0.18**  **0.17**  **0.17**  **0.06**  **0.10**  **0.19**  **0.23**  **0.37**  **0.36** | **15756**  **28125**  **28842**  **36023**  **15843**  **28364**  **35465**  **32425**  **61205**  **72850**  **27432**  **17351** | **4.67**  **2.08**  **1.53**  **1.28**  **0.91**  **0.66**  **0.10**  **0.12**  **0.13**  **0.20**  **1.83**  **10.31** | **CYP8B1 RXRA PSMB10 CPT1A VDR UCP3 CYP7A1 APOB PLTP NR1I2 APOA1 RARB** | **0.16**  **0.20**  **0.22**  **0.30**  **0.34**  **0.35**  **0.35**  **0.38**  **0.45**  **0.55**  **0.59**  **0.62** | **28716 16196** | **0.87**  **0.96** |
| **g2** | **PON1  RARB  SLC10A1 CPT1A  PPARA NRF1**  **LDLR  ACACA  PSMB10  DBI  LPIN1  CYP24A1** | **0.04**  **0.02**  **0.02**  **0.02**  **0.01**  **0.01**  **0.21**  **0.27**  **0.29**  **0.30**  **0.33**  **0.34** | **17351  27432 28661  15843 28364 28125** | **11.66**  **1.61**  **0.13**  **0.66**  **1.43**  **4.50** | **VLDLR HADHB PPARA CYP7A1 PLTP UCP3 CPT1A VDR PSMB10 APOA1 RARB NR1I2** | **0.22**  **0.22**  **0.24**  **0.25**  **0.31**  **0.31**  **0.38**  **0.40**  **0.43**  **0.44**  **0.60**  **0.63** | **17351 27432 28661 15843 28364 28125** | **0.07**  **0.12**  **0.18**  **0.26**  **0.66**  **0.75** |
| **g3** | **DBI  CYP24A1  LPIN1 ACACA  PSMB10 FAS**  **VDR  ABCB11 SLC10A1 CPT1A PON1 RARB** | **0.38**  **0.37**  **0.36**  **0.31**  **0.26**  **0.23**  **0.03**  **0.03**  **0.04**  **0.05**  **0.07**  **0.10** | **15756  28875** | **5.86**  **0.24** | **APOA1 PPARA CDKN1A CYP26A1 APOB VDR SCARB1 RXRA CPT1A PSMB10 RARB NR1I2** | **0.21**  **0.21**  **0.21**  **0.23**  **0.24**  **0.25**  **0.38**  **0.40**  **0.42**  **0.54**  **0.58**  **0.66** | **28875 15756** | **0.44**  **0.89** |
| **g4** | **ECI2**  **CBS  CYP4A14 Il2**  **ADSSL1  ELOVL6**  **MTR  ABCB1B THRSP SERPINA1A PDK4  PRG4** | **0.43**  **0.29**  **0.25**  **0.25**  **0.21**  **0.18**  **0.001 0.004**  **0.03**  **0.03**  **0.07**  **0.41** | **36023  36036  35465  32425  61204  72850 61205  28842** | **5.61**  **0.93**  **0.41**  **0.01**  **0.40**  **0.16**  **0.17**  **3.15** | **GK ABCB1B LPIN3 COX2 GLUL EIF2S3X MTR CYP4A14 PDK4 PRG4 Il2**  **FAT1** | **0.25**  **0.26**  **0.27**  **0.27**  **0.28**  **0.29**  **0.39**  **0.40**  **0.45**  **0.46**  **0.55**  **0.59** | **36036 36023 32425 61204** | **0.41**  **0.12**  **0.36**  **0.60** |
